# A Randomized, Crossover Trial Assessing Appetite, Energy Metabolism, Blood Biomarkers, and Ad Libitum Food Intake Responses to a Mid-Morning Pecan Snack vs. an Equicaloric High-Carbohydrate Snack in Healthy Volunteers with Overweight/Obesity

**DOI:** 10.3390/nu16132084

**Published:** 2024-06-29

**Authors:** John C. Peters, Jeanne Anne Breen, Zhaoxing Pan, Jacinda Nicklas, Marc-Andre Cornier

**Affiliations:** 1 Anschutz Health and Wellness Center, 12348 E. Montview Blvd., MailStop C263, Aurora, CO 80045, USA; jeanneanne.breen@cuanschutz.edu (J.A.B.); jacinda.nicklas@cuanschutz.edu (J.N.); 2 Division of Endocrinology, Diabetes and Metabolism, University of Colorado Denver, Anschutz Medical Campus, 12801 E. 17th Ave., RC1 South Rm 7103, Aurora, CO 80045, USA; 3 Department of Pediatrics, University of Colorado Denver, Anschutz Medical Campus, 13123 E. 16th Ave., B065, Aurora, CO 80045, USA; zhaoxing.pan@cuanschutz.edu; 4 Division of General Internal Medicine, University of Colorado Denver, Anschutz Medical Campus, 12801 E. 17th Ave., RC1 South Rm 7103, Aurora, CO 80045, USA; 5 Department of Medicine, Medical University of South Carolina, 96 Jonathan Lucas St., CSB 822, Charleston, SC 29425, USA; cornier@musc.edu

**Keywords:** pecans, tortilla chips, snack food, appetite, satiety, energy expenditure, blood biomarkers

## Abstract

Background: The differential effects of pecans versus other popular snack foods on appetite and blood markers of metabolism and satiety have not been well studied. This study investigated the effects of a single mid-morning snack of pecans or tortilla chips on subjective appetite, food intake, blood measures of hormones and metabolites, and resting energy expenditure. Methods: Twenty participants with overweight and obesity were enrolled in a within-participants, randomized crossover trial. Participants had indwelling catheters placed for blood sampling and were fed a standardized breakfast, followed two hours later by a 250 kcal snack of either pecans or tortilla chips, and then by a self-selected lunch. Visual analog scale (VAS) appetite measures, blood markers, and energy expenditure were taken at intervals after food consumption. Results: VAS ratings, energy, food intake and macronutrient composition did not differ between treatment conditions, but glucose and insulin were significantly more elevated after tortilla chips. Free fatty acids (FFA), triglycerides (TG), peptide YY (PYY), and glucagon-like peptide-1 (GLP-1) were higher after consuming pecans compared to tortilla chips. Conclusions: Pecan consumption improves postprandial glucose and insulin profiles which would be beneficial to individuals at risk of developing type 2 diabetes. Further studies are needed to investigate whether increased relative secretion of PYY and GLP-1 after eating pecans versus tortilla chips may affect subjective appetite and energy intake if consumed chronically.

## 1. Introduction

Pecans are one of several tree nuts for which there is a growing body of evidence describing positive health benefits, including cardiovascular health [1,2,3], appetite [4,5], and body weight management [6,7,8]. While pecans are often included in general statements about the effects of tree nuts on human physiology and health, they are less well-studied than some other tree nuts, such as almonds and walnuts. All tree nuts are relatively high in fat and energy, but there are differences in the composition of the fats present as well as their protein, carbohydrate, fiber, micronutrient, and phytochemical profiles [9,10,11], any of which may affect the physiological and behavioral responses to their consumption. Pecans are high in monounsaturated and polyunsaturated fats, low in saturated fat, protein, and carbohydrate, and high in tocopherols (particularly gamma-tocopherol), flavonoids and phytosterols [11,12,13]. As might be expected, pecans are often associated with studies reporting beneficial effects of tree nut consumption on blood lipids and lipoproteins, inflammatory markers, vascular function, and cardiovascular disease risk [3,14,15,16,17,18,19], although not all findings are consistent [20]. A smaller number of studies have directly examined the effects of pecans on cardiovascular health markers and have reported benefits largely consistent with those of almonds and walnuts [12,21,22]. The beneficial effect nuts have on blood lipids and other cardio-metabolic health markers is generally attributed to their high unsaturated to saturated fat ratio as well as their relatively high antioxidant content (e.g., from various polyphenolic compounds) [11].

Some nuts have been examined for their potential effects on body weight management. Almonds and walnuts have a reduced energy value compared to the Atwater factor estimate due to their reduced digestibility [23,24]. There are reports of enhanced satiety following nut consumption [25], although this finding is not consistent when compared to iso-caloric ingestion of other food [26]. Other studies have observed an increased thermic effect following nut consumption [27,28]. Increased satiety may be related to the high polyunsaturated fat content of nuts [29,30], while increased energy expenditure may be related either to the high content of polyunsaturated fats in nuts [31,32,33] or to their high content of flavonoids [34]. Studies have shown that when nuts are included in weight loss diets in comparison to diets without nuts, dietary compliance is improved and weight (and even weight loss) is not adversely affected [28,35,36,37,38]. Overall, despite their high fat and calorie content, nuts can be incorporated into the diet in moderation without interfering with weight loss and maintenance, while at the same time delivering benefits for cardiovascular health and diet quality [12,27,35,36,38,39,40].

To our knowledge, no studies have compared the postprandial effects of pecan snacks to other typical savory snacks of dramatically different macronutrient composition (e.g., tortilla chips) on measures of energy intake, appetite regulation (subjective evaluations of appetite, blood biomarkers of appetite), energy expenditure and substrate utilization. This may be important as diets high in carbohydrates and highly processed food have been implicated in the risk for obesity and metabolic dysregulation [41,42]. Pecans are high fat (21 g/ounce), low carbohydrate (3 g/ounce), relatively low protein (3 g/ounce) and low fiber snacks (3 g/ounce), while tortilla chips are much lower in fat (7 g/ounce), much higher in carbohydrate (19 g/ounce), with similar protein (2 g/ounce) and low fiber (1 g/ounce) contents. Both snacks are low or devoid of sugar (0–1 g/ounce). Given that snacking is a high-frequency behavior in the population and savory snacks are a large market, we conducted this study to directly compare a high-fat pecan snack to a typical high-carbohydrate savory snack. The reason we chose to compare pecans to tortilla chips instead of to other nuts is that people who are trying to lose or actively manage weight may avoid nuts because of their high fat and calorie content (for a standard weight-based serving) and choose a lower fat snack, like tortilla chips instead. Thus, it would help to better understand the effects of pecans on satiety and ad libitum food consumption in comparison to a different kind of snack that may attract weight-conscious individuals. Recent attention focused on “ultra-processed” foods and their potential contribution to weight gain and obesity [41] adds additional interest for comparing a natural, unprocessed snack like pecans, to a more highly processed snack like tortilla chips.

The major aim of this study was to characterize the metabolic, blood biomarker, subjective appetite, energy expenditure, and subsequent energy intake response to pecans when consumed as snacks compared to tortilla chips, which are comparatively much lower in fat, and much higher in carbohydrate.

## 2. Materials and Methods

### 2.1. Study Design

**Figure A1:**
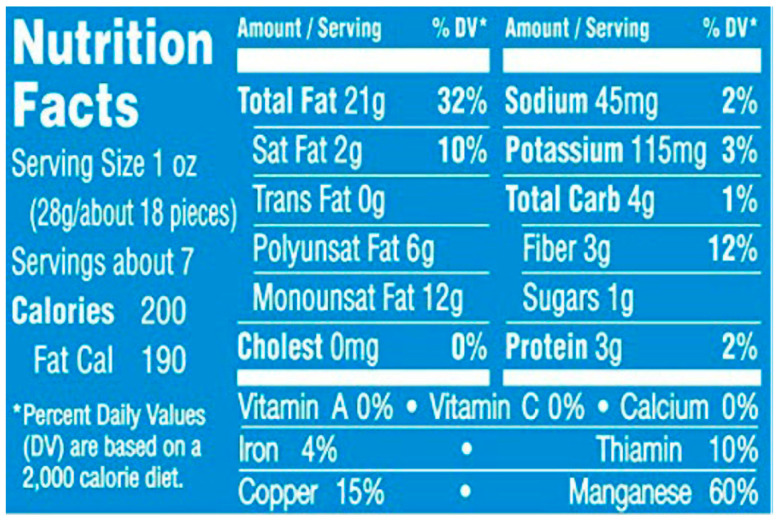
Nutrition facts panel for pecans fed in this study.

**Figure A2:**
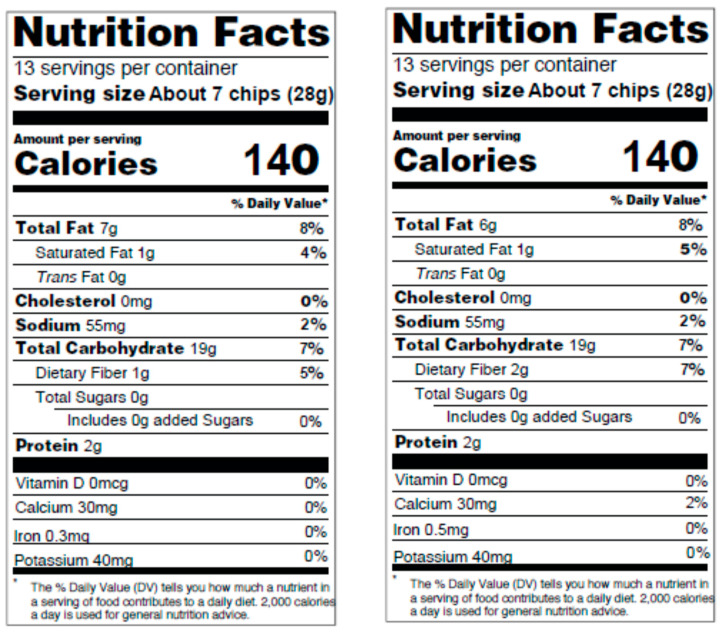
Nutrition facts panel for the two tortilla chip products used in this study.

In this study, a randomized, two-period, crossover design was used to measure the effect of different snacks on appetite, blood biomarkers, energy expenditure, and self-selected energy intake. Participants were given a standardized breakfast of 250 kcal and then either roasted and lightly salted pecans (250 kcal, 1.25 ounces, 45 mg sodium/ounce) or lightly salted tortilla chips (250 kcal, 1.79 ounces, 55 mg sodium/ounce) as a mid-morning snack. See Appendix A, Figure A1 and Figure A2 for the pecan and tortilla chip nutrition facts panels. We used two different tortilla chips of nearly identical composition for this study because one of the products became unavailable during this study, forcing us to use a different, but comparable product. The snacks were given on two separate occasions, with at least one week between them. After the snack, participants were allowed to have an ad libitum lunch, during which their food and energy intake were assessed. Blood measures of appetite hormones and metabolic markers were assessed at baseline (before breakfast), before snack, and at regular intervals after snack and lunch. Subjective appetite was assessed by visual analog scales (VAS) before breakfast, and at 15, 40, 60, 90, and 120 min intervals thereafter including post-breakfast, post-snack, and post-lunch. Metabolic rate was assessed by ventilated gas exchange before breakfast and after the snack.

### 2.2. Participants

**Figure 1:**
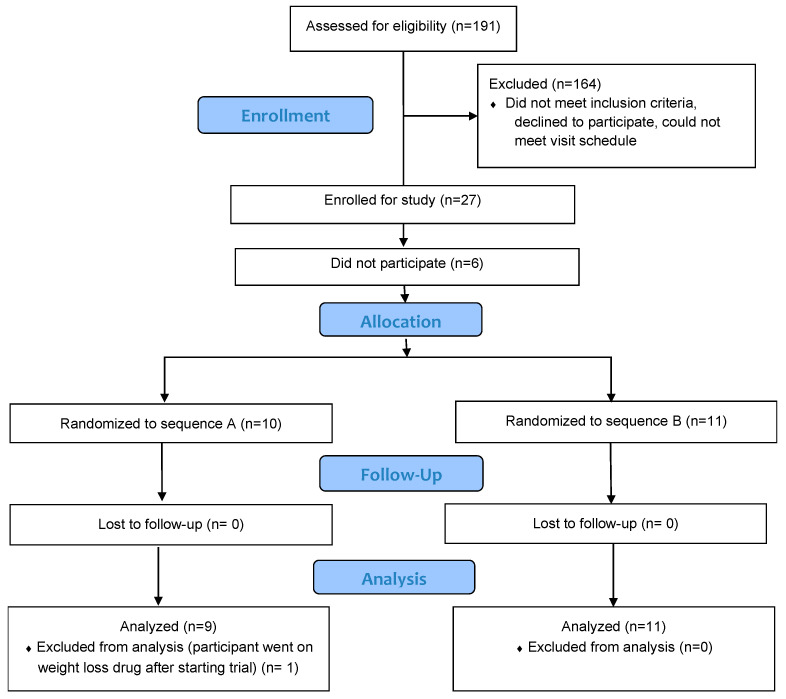
Consort Diagram.

One hundred ninety-one adult male and female volunteers aged 20–50 with a body mass index (BMI, kg/m^2^) of 27 ≤ 40 were screened for eligibility (Figure 1). Participants had to habitually eat breakfast, be willing to eat the test snacks and other foods offered, be weight stable for the last 6 months (no loss or gain of more than 3 kg in last 6 months) and be willing to consent and adhere to test procedures and schedule. People with nut allergies, uncontrolled thyroid disorders, diabetes mellitus, eating disorders, who were pregnant, or planning to become pregnant during this study, who were lactating, had uncontrolled hypertension, were actively dieting, or were participating in an intensive physical activity training regimen (>300 min/wk exercise), or with any medical history or current medication affecting appetite were excluded. Pre-menopausal women were tested in the follicular phase of their menstrual cycle if cycling regularly. Also, individuals who consumed pecans regularly (>3× per week) were excluded to avoid any potential outcome effects due to habituation. People unable to lie still with a clear hood over their head for the measurement of energy expenditure (2× at 20 min/test) were also excluded. We chose to study individuals with overweight and obesity as they are likely to be seeking dietary strategies to help manage body weight.

Of the 191 applicants, 164 did not meet the eligibility criteria and 27 were enrolled in this study. Six of those enrolled did not participate in this study. Twenty-one participants completed both treatment visits. The data for one subject were not included in the analysis as they reported starting a weight loss medication during the trial which could have affected their appetite, blood, and energy expenditure measurements.

Participants were recruited from the Anschutz Medical Campus (over 25,000 employees) and surrounding community via flyers placed on campus and in the community, study information placed on our Center website, emails sent to members of our large recruiting database accumulated from previous studies, and to the campus community and social media ads if needed. Preliminary screening was performed by telephone interview and eligible participants came to the Anschutz Health and Wellness Center (AHWC) to complete screening. Objective measurement of height, weight, BP, pulse, review of medical history, and completion of a food screening questionnaire (to document that nuts are included in their usual diet, that they are regular breakfast eaters, and to confirm that they are not habitual pecan eaters) were collected from each participant. Participants were also shown the two test snacks (bag of pecans and bag of tortilla chips) and asked to try each snack to confirm that they would be able to consume the snacks as provided on test days. Informed consent was obtained from all eligible participants.

### 2.3. Protocol

#### 2.3.1. Screening Visit

Baseline measures and washout week: Participants were asked to refrain from exercise and alcohol consumption the day before each test day and until after discharge on test days and were asked to not consume/use any food, caffeine, or marijuana for 12 h before testing (i.e., from 7 p.m. the night before test days). Further, participants were asked to abstain from any nut consumption during the one week before each test day.

#### 2.3.2. Testing Visits

Test day visits and preparation: Participants were given food items to prepare and consume a standard dinner between 5 and 7 p.m. the night before each test day. The meal contained approximately 35% of the estimated total daily energy intake, 15% protein, 30% fat, and 55% carbohydrate. Participants were asked to fast after 7 p.m. the evening before each test day. On test days, participants reported to the clinic early in the morning after an overnight fast and underwent an assessment of resting metabolic rate (RMR; 20 min. assessment following a 30 min rest). An indwelling intravenous catheter was then placed in one arm for blood sampling throughout the test day. A baseline blood sample was drawn, and subjective measures of appetite were obtained using visual analog scales (VAS). They then received a standardized breakfast providing approximately 266 total kcal, consisting of 18% protein, 30% fat, and 52% carbohydrate (as % of calories). The content of the breakfast was Eggbeater scrambled eggs, whole wheat bread with butter, orange juice, and 12 ounces of water. They were asked to consume the entire breakfast in 15 min. VAS measures of hunger, fullness, and prospective consumption [43] were completed at time zero and 15, 40, 60, 90, and 120 min following breakfast until the test snacks were provided mid-morning. A pre-snack blood sample was drawn, a VAS scale was completed, and the test snacks (250 kcal; 1.25 ounces of pecans, and 1.79 ounces of tortilla chips) were consumed in their entirety (15 min to consume; 8 ounces of water provided). REE (20 min measure) was assessed immediately after snack consumption. VAS appetite scales and blood were completed/drawn at 15, 40, 60, 90, and 120 min following snack consumption, with the last recording/sample occurring just before consumption of an ad libitum lunch meal.

The ad libitum lunch meal consisted of a variety of different foods offering approximately 1843 kcal of energy with an overall composition of approximately 15% protein, 55% carbohydrate, and 30% fat. Twelve ounces of water and one can each of regular Pepsi and Diet Pepsi were provided as the beverages. Participants were given 30 min for lunch and instructed to eat what they wanted. They could request more of any food. This design neither restricted intake nor encouraged overconsumption. Blood samples and VAS appetite ratings were taken/completed at 15, 40, 60, 90, and 120 min from the start of lunch. Participants were then discharged.

### 2.4. Outcome Measures

Energy and macronutrient intake at lunch: Ad libitum energy intake and macronutrient composition consumed at lunch were assessed by weighing all food served to participants and weighing any remaining food after the meal and calculating energy and nutrient intake based using the ProNutra™ (version 3.6.0.1) nutrition software.

Blood measures: The following analytes were determined using the Olympus Chemistry Analyzer, Beckman AU 480, Beckman Coulter, Brea, CA, USA. Plasma glucose was determined by the hexokinase method. Serum insulin was determined by a chemiluminescence immunoassay using the Access^®^, Ultrasensitive Insulin Reagent. Serum free fatty acids (FFA) and plasma triglycerides (TG) were measured spectrophotometrically using Fisher Scientific reagents (Thermo Fisher Scientific, Waltham, MA, USA). Plasma total glucagon-like peptide-1 (GLP-1) was measured by ELISA kit, Mercodia, Uppsala, Sweden. Plasma peptide YY (PYY), leptin, and ghrelin were measured by radio-immunoassay kit, Millipore Sigma, Burlington, MA, USA.

All serum and plasma samples were stored at −70 °C until analysis. Homeostatic model assessment of insulin resistance (HOMA-IR) was calculated from fasting glucose and insulin (HOMA-IR = Glucose (mg/dL) × Insulin (μU/L)/405) [44].

Energy expenditure: Resting (RMR) and post-meal resting energy expenditure (REE) were measured by standard indirect calorimetry using the ventilated hood technique (Parvo Medics Truemax 2400, Salt Lake City, UT, USA). This was performed at baseline (before breakfast) and after the mid-morning snack. The baseline measure (RMR) was taken after 30 min of supine quiet rest. Post-snack measures of energy expenditure (REE) were taken when participants were at rest (sitting, before laying down for the measure) during their stay in the test facility. Data from 3 min to 18 min (out of the 20 min measure) were used in the analysis as this time yielded the most stable readings (eliminating effects of movement as participants were getting comfortable).

Appetite: During the study day, participants marked visual analog scales (hunger, fullness, desire to eat, prospective consumption) administered at baseline, and at 15, 40, 60, 90, and 120 min after breakfast, snack, and lunch. Subjective feelings were rated on a 100 mm horizontal line preceded by the questions: “How hungry are you right now?” and anchored on the left by “not at all hungry” and by “extremely hungry” on the right; Fullness was queried by “How full do you feel right now?” anchored by “not at all…” and “extremely…”; Desire to eat was queried by, “How strong is your desire to eat right now?” anchored by “not at all…” and “extreme…; and prospective consumption was queried by, “how much food do you think you can eat right now?” with anchors of “not (much) at all” to “extremely/an extreme amount”. Participants marked a vertical line on the 100 mm horizontal line to indicate their feelings in response to the questions.

The snack, meal, and sampling schedules are shown in Table 1.

### 2.5. Statistical Analysis

Randomization and sample size: Participants were randomized by a computer algorithm. There were no previous studies to estimate the potential effect size of pecans as compared to tortilla chips on subjective appetite or food intake in this study. We determined the sample size to ensure at least 80% power at a 5% significance to detect a clinically meaningful effect between the two snack conditions for subjective appetite and food intake, and to ensure this study would have a Williams design ([45] even number of participants). No adjustment of type 1 error for multiple outcomes was used because of the exploratory nature of this study. Mean and SD for energy intake at lunch were assumed to be 705 kcal and 299 kcal, respectively, taken from a recently completed study of 44 participants of similar age and BMI range performed in Dr. Cornier’s lab [46]. Mean and SD for VAS measures were taken from a weight loss study performed by our group [47]. If we assume that the SD is the same for two visits and a typical correlation between measures at two different visits is 0.75, then using the NQuery module of power analysis for 2 × 2 crossover design, a sample size of 10 per sequence group (total of 20) is required to have 84% power at 5% significance to detect effect sizes of 7–8 mm on a 100 mm scale for hunger and fullness (which have been meaningful in previous studies from our group, e.g., [46,47]), and 142 kcal for energy intake at lunch. At least 24 participants needed to be enrolled to allow for 20% attrition.

Analysis of outcome variables: Separate analyses were conducted for outcome measures evaluated over post-breakfast, post-snack, and post-lunch intervals. Using the trapezoidal rule, individual repeated measures of laboratory and VAS variables over each follow-up interval were, respectively, summarized using the area under the curve (AUC) and AUC increment (iAUC), mean, maximum, and nadir over the corresponding interval. These summary variables for repeated outcome measures and outcomes without repeated measures such as energy intake were analyzed using the Linear mixed effects model (LMM) with random subject effect to test the difference in the outcome between two experimental conditions. The model consists of sequence group (pecan-tortilla chips or chips-pecan), period (1st or 2nd visit), and treatment (pecan or tortilla chips) as fixed effects. The effect of pecans as compared to tortilla chips was assessed by testing the between-condition difference in the least square means from the model. In addition, we modeled the repeated measure data in the raw scale by introducing a time effect and interaction term of time by treatment in the abovementioned LMM model, to delineate the profiles of trajectory and assess the difference between two experimental conditions. Analyses from both approaches produced consistent results. The appropriateness of the normal assumption of the LMM model was examined by graphically reviewing residual plots. The ninety-percent confidence interval was estimated from the LMM model. *p*-values < 0.05 were deemed statistically significant. No adjustment for multiple outcomes as well as multiple a priori comparisons for a given outcome were applied. SAS 9.4 software (SAS Institute Inc., Cary, NC, USA) was used for all the analyses. Of particular note, there is a participant whose glucose response after the pecan snack was 3 to 4 times higher for several repeated measures as compared to the rest of the participants. Sensitivity LMM analysis excluding this participant produced opposite response patterns and statistical testing results. Therefore, we reported glucose results from the analyses excluding this influential participant.

## 3. Results

### 3.1. Participant Characteristics

Fourteen female and six male participants completed this study as shown in Table 2. The mean age of participants was 35.8 years and the mean body mass index (BMI, kg/m^2^) was 30.9 with a range of 27.2–39.2, encompassing the study inclusion criteria of 27–40. Baseline blood markers (glucose, insulin, FFA, TG, ghrelin, peptide YY, GLP-1, and leptin were all within normal ranges and were not significantly different between visits. Participants were also not insulin resistant with HOMA-IR values ≤ 2.

### 3.2. Subjective Appetite Measures

**Figure A3:**
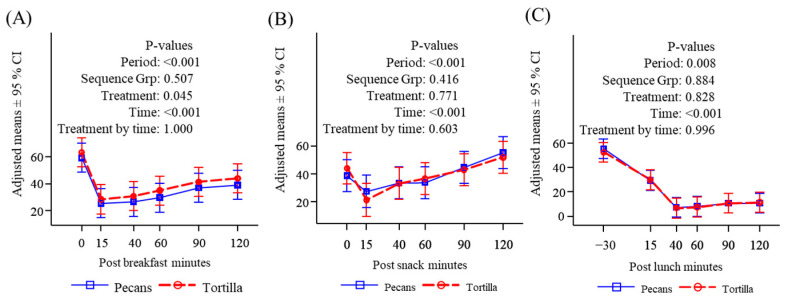
How hungry are you right now? Visual analog scale appetite scores on a 0–100 mm scale. (**A**) Before and after breakfast; (**B**) before and after snack; (**C**) before and after lunch.

**Figure A4:**
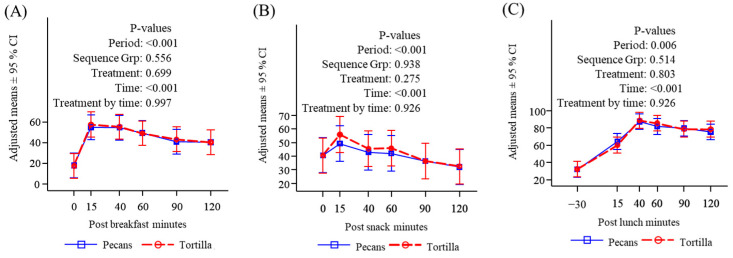
How full do you feel right now? Visual analog scale appetite scores on a 0–100 mm scale. (**A**) Before and after breakfast; (**B**) before and after snack; (**C**) before and after lunch.

**Figure A5:**
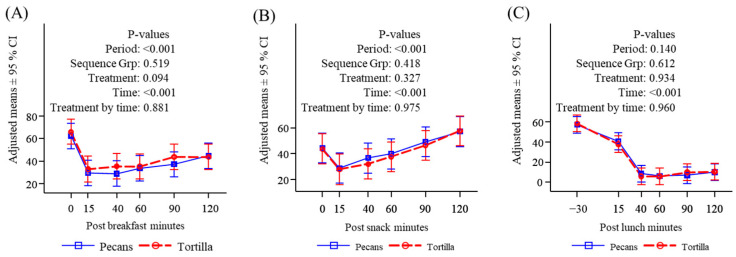
How strong is your desire to eat right now? Visual analog scale appetite scores on a 0–100 mm scale. (**A**) Before and after breakfast; (**B**) Before and after snack; (**C**) Before and after lunch.

**Figure A6:**
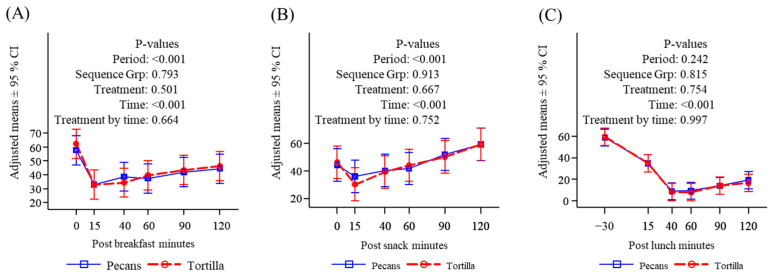
How much do you think you could eat right now? Visual analog scale appetite scores on a 0–100 mm scale. (**A**) Before and after breakfast; (**B**) Before and after snack; (**C**) Before and after lunch.

Subjective reports of hunger, fullness, desire to eat, and prospective consumption were not significantly different between the tortilla chip and pecan conditions before and after breakfast, snack, and lunch meals (see Appendix A, Figure A3, Figure A4, Figure A5 and Figure A6). Visual analog responses for each of the measures followed the expected patterns of change following food consumption, with hunger, desire to eat, and prospective consumption declining and then increasing before the next eating occasion, and fullness increasing and then declining before the next meal.

### 3.3. Breakfast, Snack, and Self-Selected Lunch Consumption

**Figure 2:**
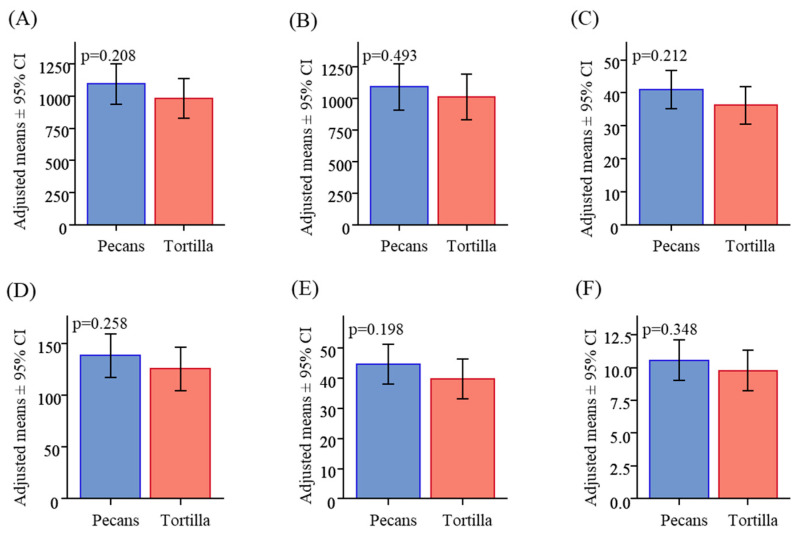
Nutritional composition of self-selected lunch. (**A**) Energy consumed (kcal); (**B**) weight of food consumed (g); (**C**) protein consumed (g); (**D**) carbohydrate consumed (g); (**E**) fat consumed (g); (**F**) fiber consumed (g).

Participants consumed on average all of the breakfast and the entire snack as instructed. There were no differences in breakfast or snack intake as a function of condition (pecan or tortilla chip snack) or of visit order. Likewise, there were no significant differences in energy intake, weight of food consumed, or macronutrient composition at lunch between the two snack treatment conditions (Figure 2A–F).

### 3.4. Plasma Metabolites and Appetite Hormones

#### 3.4.1. Glucose

**Figure 3:**
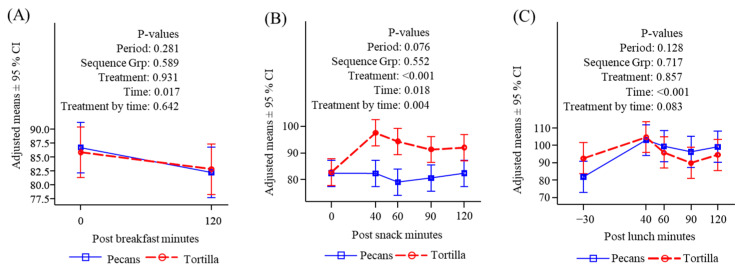
Plasma glucose (mg/dL) response to meals. (**A**) Before and after breakfast; (**B**) before and after snack; (**C**) before and after lunch.

There were no differences in participants’ glucose levels either before or after the standardized breakfast on either testing day (Figure 3A). Consumption of tortilla chips caused a significantly greater increase in blood glucose at all time points compared to pecans (Figure 3B), and a similar significant treatment difference was observed when examined as an area under the response curve, which was performed to account for any potential effect of multiple comparisons. Glucose responses following lunch were not different between the two treatments (Figure 3C).

#### 3.4.2. Insulin

**Figure 4:**
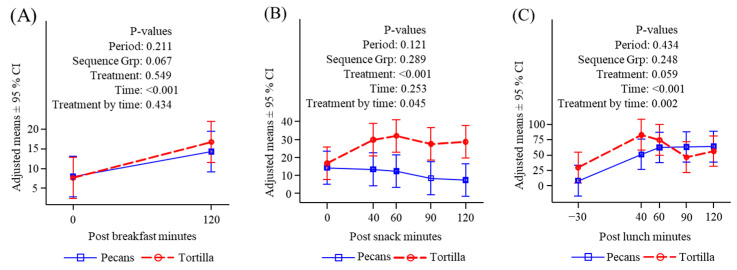
Serum insulin (μU/mL) response to meals. (**A**) Before and after breakfast; (**B**) before and after snack; (**C**) before and after lunch.

Insulin responses to consumption of the standardized breakfast did not differ on the two treatment days (Figure 4A). Consumption of tortilla chips provoked a significant increase in insulin both compared to time zero and to the pecan response. The pecan snack did not increase insulin and there was a downward trend over time since breakfast. (Figure 4B). There were no significant differences in insulin following lunch between either treatment, and insulin increased under both conditions (Figure 4C). The initial values were nearly significantly greater for the chip condition reflecting a carryover from the mid-morning snack effect.

#### 3.4.3. Free Fatty Acids

**Figure 5:**
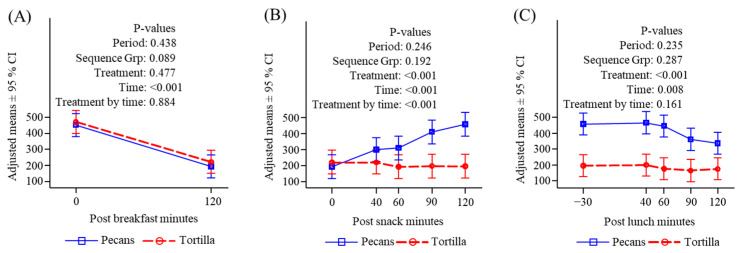
Serum free fatty acids (μmol/L) response to meals. (**A**) Before and after breakfast; (**B**) before and after snack; (**C**) before and after lunch.

Free fatty acids declined in the post-breakfast period and there were no differences between treatment conditions (Figure 5A). Pecan consumption caused a significant rise in FFA levels both from time zero and compared to tortilla chips (Figure 5B). Following lunch, there was a significant decline in FFA in the pecan condition starting from a high-level carryover from the snack and remaining above the level seen in the chip condition throughout the measurement period (Figure 5C).

#### 3.4.4. Triglycerides

**Figure 6:**
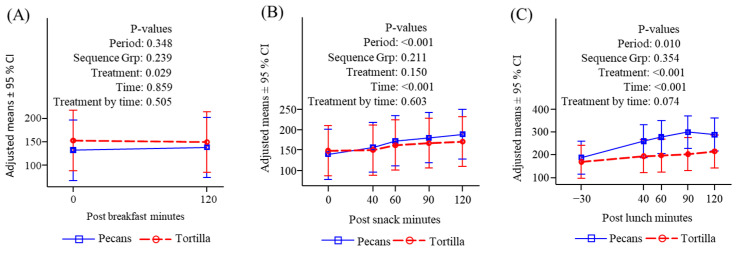
Plasma triglycerides (mg/dL) response to meals. (**A**) Before and after breakfast; (**B**) before and after snack; (**C**) before and after lunch.

Triglycerides were significantly greater in the chip condition compared to the pecan condition before and after breakfast (Figure 6A); however, they did not differ at the start of the mid-morning snack and were not different between conditions following the snack (Figure 6B). After lunch, triglycerides significantly increased in the pecan condition when compared to the chip condition and were still greater compared to the chip treatment when the measurement period stopped (Figure 6C).

#### 3.4.5. Ghrelin

**Figure 7:**
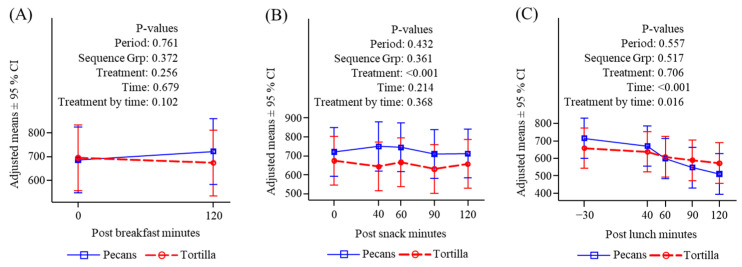
Plasma ghrelin (pg/mL) response to meals. (**A**) Before and after breakfast; (**B**) before and after snack; (**C**) before and after lunch.

There was no difference in ghrelin response to the standardized breakfast between the two conditions (Figure 7A). Ghrelin was significantly greater following the pecan snack consumption compared to the tortilla chips although the increase compared to time zero was not significant (Figure 7B). There were no differences between treatment conditions in ghrelin levels following lunch and there was a meal-related decline throughout the measurement period (Figure 7C).

#### 3.4.6. Peptide YY

**Figure 8:**
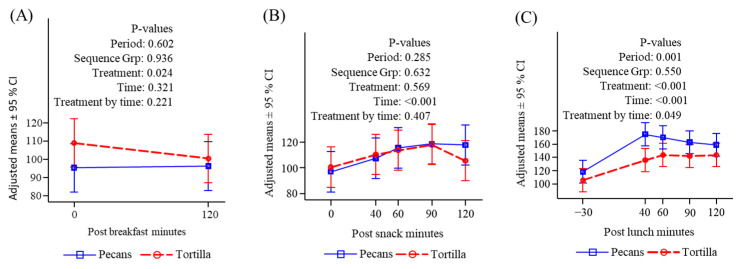
Plasma Peptide YY (pg/mL) response to meals. (**A**) Before and after breakfast; (**B**) before and after snack; (**C**) before and after lunch.

Peptide YY was greater at baseline in the chip condition compared to the pecan condition and declined after breakfast and was not different between conditions two hours later (Figure 8A). Levels of PYY increased after lunch and were not different between treatment conditions (Figure 8B). Following lunch, PYY levels increased in both conditions, and the increase was significantly greater in the pecan condition, possibly owing to a carryover from the effect of the pecan snack on circulating FFA, which are potent stimulators of PYY (Figure 8C).

#### 3.4.7. GLP-1

**Figure 9:**
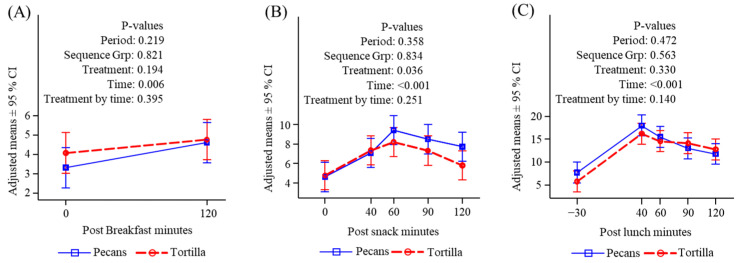
Plasma GLP-1 (pmol/L) response to meal. (**A**) Before and after breakfast; (**B**) before and after snack; (**C**) before and after lunch.

GLP-1 increased after breakfast, and there was no difference between the two snack conditions (Figure 9A). There was a significant increase in GLP-1 following snack consumption and there was a greater rise following the pecan treatment compared to tortilla chips (Figure 9B). Following lunch, GLP-1 increased significantly, and there was no difference between the treatment conditions (Figure 9C).

### 3.5. Resting and Postprandial Energy Expenditure

**Figure 10:**
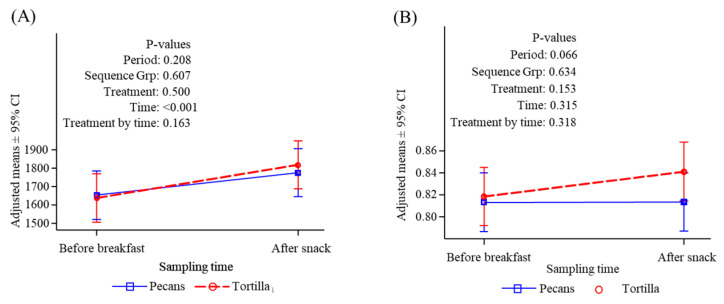
Resting and post-snack energy expenditure (REE, kcal/d), and respiratory quotient (RQ). (**A**) REE, before breakfast and after snack; (**B**) RQ, before breakfast and after snack.

Resting energy expenditure was not different at baseline (before breakfast) under either snack condition and there were no differences between conditions in the response to consumption of the standardized breakfast (Figure 10A), whether expressed as an average of energy expenditure over the measurement period or as an area under the curve. Resting energy expenditure increased from baseline by 7.4% in the pecan condition and by 10.9% in the tortilla chip condition, although these were not significantly different. The response measured after snack consumption represents both the residual response to breakfast as well as the response to snack. Because the breakfast was a fixed composition, any difference in response after the snack should reflect the effect of the particular snack fed. The RQ response to breakfast and snack was also not significantly different between conditions (Figure 10B), although the mean RQ increased following the tortilla chip snack while there was no change after consuming pecans.

## 4. Discussion

In this study, we characterized the acute appetitive and metabolic responses to two common snack foods with quite different nutritional compositions, pecans, which are high in fat and low in carbohydrate, and tortilla chips, which are high in carbohydrate and moderate in fat content. Both snacks had similar protein content. Tree nuts, like pecans, and tortilla chips are popular snacks, and it was of interest to understand if one or the other is more effective at reducing hunger and appetite as well as to document their effects on markers of metabolic health and disease risk. This may be especially important for individuals with overweight and obesity who may be at risk of developing type 2 diabetes [48].

We found that a single exposure to a 250 kcal isocaloric snack of either tortilla chips or pecans did not differentially affect subjective measures of hunger, fullness, desire to eat, or prospective consumption. The treatments also did not affect the amount of food, energy, or macronutrients consumed at a self-selected lunch meal. We also observed high energy intake at lunch (approximately 1000 kcal) after participants had already consumed 510 kcal at breakfast and snack. Our sample consisted of mainly women (14/20) and their daily calorie intake to maintain body mass would be approximately 2100 kcal for light exercise. Thus, leaving room for only 600 kcal for the rest of the day. The high intake at lunch is typical when a large self-selection assortment is offered [43] and this test condition may obscure any small difference in lunch intake that might result from the different snack treatments.

The pattern of subjective appetite responses following the meals and snacks was consistent with what would be expected following the consumption of food energy [49]. The lack of a differential effect of the two mid-morning snacks on subjective appetite and intake at the subsequent lunch meal may not be surprising since the caloric load consumed was the same. However, the weight and volume of the tortilla chips were significantly greater than that of the pecans, owing to their lower caloric density, 4.99 kcal/g for chips vs. 7.14 kcal/g for pecans. It has been observed that the weight, volume, and energy density of a food can affect satiety and satiation independent of the energy content [50,51,52,53,54], suggesting that the smaller weight and volume of the pecan snack could have been less satiating than the larger amount of tortilla chips, although this was not the case.

It is also possible that familiarity, previous experience, and expectation of particular subjective feelings following consumption of the two snacks may have dominated the response rather than any effect of the snack composition on physiological drivers of satiety and food intake. Our group has shown that despite no variable changes in measures of appetite with over or underfeeding, significant changes are seen in the neuronal responses to food-related cues [55,56]. Other investigators have found that intravenous co-infusion of the satiety hormones GLP-1 and PYY caused a significant reduction in food intake but did not affect subjective measures of appetite [57]. Intravenous infusion of the appetite hormones versus a food-provoked increase would eliminate any expected changes in subjective appetite ratings based on the appearance, amount, and any previously experienced appetite sensations, perhaps explaining why there was no effect. Expectations about satiety and satiation are conditioned upon multiple exposures to a food over time [58], and a single exposure under experimental conditions would likely not be expected to provoke a differential response to the two snacks. Furthermore, subjective ratings of appetite have not been shown to be good predictors of food or energy intake [43].

Given that we observed a significant increase in GLP-1 after pecan snack intake and an increase in PYY following lunch intake compared to the tortilla chip condition suggests that a rise in these satiety hormones after a single exposure may not be sufficient to affect subjective feelings of fullness and reduced hunger. It may be necessary to have repeated exposure to these snack foods over time so that changes in the satiety hormones are consistently associated with changes in internal feelings of hunger and satiety, affecting expectations and ultimately VAS appetite scores.

Other authors have found a similar discordance between changes in blood satiety hormones and subjective appetite following a single nut-enriched meal (walnuts) compared to a nut-free reference meal [59]. By contrast, feeding pecan-enriched diets for 8 weeks was associated with reduced overall appetite, desire to eat, prospective consumption, and greater fullness compared to a nut-free diet [4], suggesting that repeated exposure to the effects of different foods can produce changes in subjective appetite measures.

Although subjective hunger and appetite were not differentially affected by the two snacks, there were significant differences in metabolic markers and appetite hormones. We observed significant differences in the responses of glucose, insulin, fatty acids, and triglycerides to consumption of the snacks, which were consistent with their carbohydrate and fat composition. Tortilla chips provoked a more marked increase in glucose and insulin than did pecans owing to the much greater carbohydrate content of the snack fed (34 g for tortilla chips vs. 5 g for pecans). Tortilla chips have a medium Glycemic Index of approximately 60 [60], and given the 34 g serving, the glycemic load would be approximately 20.4, which would be considered a high glycemic snack [61]. The rise in insulin following the chip snack carried over to lunch two hours later while insulin declined slightly after the pecan snack. The greater elevation of glucose and insulin after consuming the tortilla chip snack might suggest that such high-carbohydrate snacks could be a risk factor in the development of insulin resistance and type 2 diabetes [62].

The pecan snack was associated with a prolonged rise in serum FFA over the snack and lunch period that was not observed after the tortilla chip snack, where FFA were unchanged. The rise in FFA after the pecan snack was likely due to the lack of insulin stimulation, which would eliminate insulin action to suppress lipoprotein lipase and hence the release of FFA from TG hydrolysis [63,64]. The consecutive consumption of the tortilla chip snack and lunch provoked a prolonged rise in insulin across the 4 h of measurement (from the start of snack to the end of lunch), effectively suppressing TG hydrolysis and FFA release during the entire period.

The pecan condition was also associated with a significant rise in TG (compared to tortilla chips) beginning late after snack consumption and becoming significant 40 min after the start of lunch. This rise was likely the combined effect of consuming the high-fat snack (26 g fat) plus an additional amount of fat (approximately 40 g) at lunch. Peak blood TG levels occur approximately 4–5 h after a high-fat meal [65], timing which is consistent with the peak TG levels occurring approximately 3.5 h after consumption of the high-fat pecan snack.

Ghrelin is often thought of as the hunger hormone, with its level being elevated before a meal [66], and declining after food consumption [67]. Plasma ghrelin levels were significantly greater following pecan consumption compared to tortilla chips, suggesting that the chips suppressed hunger to a greater extent than pecans. This is consistent with other studies demonstrating that a high-carbohydrate meal is more effective at suppressing ghrelin than a high-fat meal [68]. Despite this finding, VAS hunger ratings were not different between the two snack conditions. Other studies have found that ghrelin or changes in ghrelin do not predict or associate with hunger ratings [69]. In addition, ghrelin responses to the lunch meal were not different and showed the expected decline following food intake [67,70], and there were no differences in total food and energy intakes.

PYY is a satiety hormone released from intestinal enteroendocrine L cells in response to food intake, with levels rising within 15 min and reaching a plateau within 1–2 h after a meal [71]. Dietary protein, fat, and to a lesser extent carbohydrate stimulate PYY secretion [72,73], We observed the expected rise in PYY after both snack and lunch consumption in both treatment conditions. However, the increase in PYY was greater after lunch in the pecan condition which reflects a carryover from a rise occurring late after the snack (Figure 8B,C). The pattern of PYY elevation in the pecan condition followed the pattern of TG increase (Figure 6B,C), which may have resulted from the absorption of fat contained in both the snack and lunch. This response is consistent with the known effects of dietary fat on stimulating PYY secretion [74,75].

GLP-1 is a satiety hormone also secreted by the L cells of the distal gastrointestinal tract and its most potent dietary secretagogues are carbohydrates and fat [76,77]. We observed the expected increase in GLP-1 following food ingestion, with a significantly greater rise following the pecan snack compared to the tortilla snack between 60 and 120 min (Figure 9B). The response to the lunch meal was not different between conditions (Figure 9C), consistent with the observation that the nutrient composition of the self-selected lunch was not different between conditions. Despite the significant difference in GLP-1 level after the snack, there was no difference in any of the subjective appetite measures, a finding consistent with other studies showing changes in appetite hormones without associated changes in subjective appetite [4,57].

Resting energy expenditure was increased by breakfast and snack intake as would be expected due to the thermic effect of food [78]. The increase was not significantly different between the two snack conditions, although the mean rise was greater after tortilla chips (approximately 11%) compared to pecans (approximately 7%). Carbohydrates are known to stimulate the thermic effect more than fats [78] and our findings are consistent with this expectation. Other investigators have reported that 8 weeks of consumption of a pecan-enriched diet led to significant increases in the thermic effect of food and fat oxidation following a high-fat test meal compared to values at baseline or compared to a control diet [79]. It is possible that long-term consumption of pecans may increase the thermic effect in response to fat, but it still may not exceed the effect of a high-carbohydrate treatment. The RQ in our study was also not significantly different between treatment conditions either after snack or lunch, although the RQ was numerically greater after the chips compared to the pecans, reflecting increased oxidation of the absorbed carbohydrates from the high-carbohydrate tortilla chips.

This study has several strengths, including the crossover design with each subject being exposed to both snack treatments, and using a wide range of measures, both subjective appetite, objective food intake, blood measures of important metabolites, appetite hormones, and postprandial energy expenditure. It is the first comparison, to our knowledge, of the appetitive and metabolic effects of two commercially available snack foods that are widely consumed. Finally, the participants all had overweight and obesity which is a population that might be at greater risk for undesirable metabolic effects of different snack foods.

This study also had several limitations, including the largely female participant population, making it difficult to ascertain any sex differences in treatment response due to power limitations. We did not document and control for habitual smoking or exercise behaviors, factors that could affect appetite and metabolic measures. Also, the single-meal challenge nature of the treatment was insufficient to allow conditioning of the physiological response to the treatments to the subjective feelings experienced.

## 5. Conclusions

A single exposure to an isocaloric snack of either pecans or tortilla chips did not differentially affect subjective hunger, satiety, desire to eat, or prospective consumption. The treatments also did not differentially affect intake at a self-selected lunch. However, there were significant differential effects on blood markers of satiety and energy metabolism, with the chip treatment stimulating a prolonged elevation of glucose and insulin, while the pecan treatment caused little if any increase. Pecans also significantly increased PYY and GLP-1 compared to tortilla chips, which might lead to changes in subjective appetite and energy intake over time. Further studies are needed to explore this possibility. The more favorable post-ingestive profile of glucose and insulin after consuming pecans suggests that they may be beneficial to individuals at risk of developing metabolic dysfunction and type 2 diabetes.

## Figures and Tables

**Table 1:** Test snack, meal feeding, and outcomes assessment schedule.

Time (min)	−30	0	15	40	60	90	120	15	40	60	90	120	15	40	60	90	120
Meal		B’fast					Snack					Lunch					
Glucose		X					X		X	X	X	X		X	X	X	X
Insulin		X					X		X	X	X	X		X	X	X	X
FFA		X					X		X	X	X	X		X	X	X	X
TG		X					X		X	X	X	X		X	X	X	X
Leptin		X															
Ghrelin, PYY, GLP1		X					X		X	X	X	X		X	X	X	X
Appetite ratings		X	X	X	X	X	X	X	X	X	X	X	X	X	X	X	X
Calorimetry	X							X									

**Table 2:** Participant characteristics.

Characteristic	(N = 20)
Sex n, (%)	Female 14 Male 6
Age, mean (SD ^1^, range) Age, median	35.8 (8.6; 24–51) 38.5
Ethnicity n, (%)	Hispanic/Latino 5 (25) Non-Hispanic/Latino 15 (75)
Race n, (%)	White 13 (65) Black or AA 2 (10) Asian 3 (15) Other 2 (10)
Height (cm) mean (SD)	165.0 (9.3)
Body weight (kg) mean (SD)	84.7 (15.9)
BMI ^2^ (kg/m^2^) mean (SD, range) BMI median	30.9 (3.3, 27.2–39.2) 29.8
Leptin (ng/mL) mean (SD); Visit 1; Visit 2	37.4 (27.5) 34.9 (22.3)
Glucose (mg/dL) mean (SD); Visit 1 Visit 2	85.8 (6.6) 87.3 (12.7)
Insulin (µU/mL) mean (SD); Visit 1 Visit 2	7.9 (6.0) 6.9 (5.3)
HOMA-IR mean (SD); Visit 1 Visit 2	1.7 (1.4) 1.6 (1.4)
FFA (µmol/L) mean (SD); Visit 1 Visit 2	443.0 (191.0) 470.8 (161.8)
Triglycerides (mg/dL) mean (SD); Visit 1, Visit 2	143.9 (170.2) 133.9 (118.8)
Ghrelin (pg/mL) mean (SD); Visit 1 Visit 2	697.7 (289.2) 694.7 (319.0)
Peptide YY (pg/mL) mean (SD); Visit 1, Visit 2	105.9 (33.1) 98.5 (28.8)
GLP-1 (pmol/L) mean (SD); Visit 1 Visit 2	4.6 (4.3) 8.1 (19.7)

^1^ SD, standard deviation; ^2^ BMI, body mass index (kg/m^2^).

## Data Availability

Data described in this article can be requested by any qualified researcher and will be provided upon reasonable request.

## Appendix A

Subjective appetite measures.
